# Best Practices for the Nutritional Management of Infantile-Onset Lysosomal Acid Lipase Deficiency: A Case-Based Discussion

**DOI:** 10.3390/nu18020233

**Published:** 2026-01-12

**Authors:** Fiona J. White, Javier de las Heras, Celia Rodríguez-Borjabad, Simon A. Jones, Alexander Y. Kim, Jenna Moore, Florian Abel, Laura Frank, Rosie Jones, Suresh Vijay

**Affiliations:** 1 Genomic Medicine, St. Mary’s Hospital, MFT, University of Manchester, Manchester M13 9WL, UK; fionajwhite45@gmail.com (F.J.W.); simon.jones@mft.nhs.uk (S.A.J.); 2 Biobizkaia Health Research Institute, 48903 Barakaldo, Spain; 3 Division of Pediatric Metabolism (CIBER-ER), Cruces University Hospital, 48903 Barakaldo, Spain; 4 Department of Pediatrics, University of the Basque Country (UPV/EHU), 48940 Leioa, Spain; 5 Unitat de Nutrició i Unitat de Medicina Vascular i Metabolisme, Hospital Sant Joan de Reus, 43204 Reus, Spain; 6 Division of Genetics, Department of Medicine, Johns Hopkins All Children’s Hospital, St. Petersburg, FL 33701, USA; ayk@jhmi.edu; 7 Nutritional Services, Johns Hopkins All Children’s Hospital, St. Petersburg, FL 33701, USA; 8 Department of Global Medical Affairs, Alexion, AstraZeneca Rare Disease, Boston, MA 02210, USA; florian.abel@alexion.com; 9 Department of Metabolics, Alexion, AstraZeneca Rare Disease, Boston, MA 02210, USA; 10 Dietetics Department, Birmingham Women’s and Children’s Hospitals NHS Foundation Trust, Birmingham B15 2TG, UK; rosiejones1@nhs.net; 11 University Hospitals Birmingham NHS Foundation Trust, Birmingham B15 2GW, UK

**Keywords:** dietary substrate reduction, substrate reduction therapy, early diagnosis, early treatment, enzyme replacement therapy, infantile-onset lysosomal acid lipase deficiency, LAL-D, malabsorption, minimal lipid intake, sebelipase alfa, Wolman disease

## Abstract

Infantile-onset lysosomal acid lipase deficiency (LAL-D) (Wolman disease, historically) is a rare inherited, rapidly progressive disorder caused by pathogenic variants in the *LIPA* gene, which encodes the enzyme LAL. LAL is essential for the metabolism of cholesteryl esters and triglycerides. LAL deficiency leads to the accumulation of cholesteryl esters and triglycerides within the lysosomes, macrophages, and parenchymal cells in most tissue types, including those in the liver, gastrointestinal tract, and lymph nodes but excluding the central nervous system. Infants with rapidly progressive LAL-D present with gastrointestinal disturbance, adrenomegaly with calcification, hepatosplenomegaly, growth failure due to malabsorption, and systemic inflammation. If untreated, rapidly progressive LAL-D typically leads to death within the first year of life. Treatment takes the two-pronged approach of sebelipase alfa, a human lysosomal acid lipase enzyme replacement therapy (ERT) that improves lipid metabolism, combined with nutritional management. Dietary substrate (lipid) reduction, known as substrate reduction therapy, is essential for optimal management in LAL-D. Following a nutritional plan and managing gastrointestinal disturbances together reduce systemic inflammation and improve growth, gut function, liver health, quality of life, and survival in patients with infantile-onset LAL-D. A multidisciplinary specialized team is necessary to manage the highly complex, multisystemic conditions in these patients. Nutritional management of LAL-D has evolved with increasing experience with the clinical management of ERT-treated infantile-onset LAL-D. A review of guidance for best practice nutritional management is needed. This narrative review aims to provide updated recommendations and guidance for the optimal nutritional management of infantile-onset LAL-D.

## 1. Introduction

Lysosomal acid lipase deficiency (LAL-D) is a rare, hereditary, and progressive autosomal recessive lipid metabolism and lysosomal storage disorder associated with pathogenic variants in the *LIPA* gene, which encodes for the enzyme LAL [1,2,3]. LAL is essential for the breakdown of cholesteryl esters and triglycerides [1,2,3,4]. LAL-D leads to an accumulation of cholesteryl esters and triglycerides within the gastrointestinal tract, liver, adrenal glands, and macrophages; notably, the exception is the central nervous system due to the protective effects of the blood–brain barrier [1,2,3,4]. Dysfunction caused by the systemic accumulation of lipids can be life-threatening if not appropriately treated [5,6]. In rapidly progressive, infantile-onset LAL-D (Wolman disease, historically), patients usually first present with malabsorption and growth failure, and untreated infantile-onset LAL-D is typically fatal within the first year of life [2,5,6].

**Figure 1:**
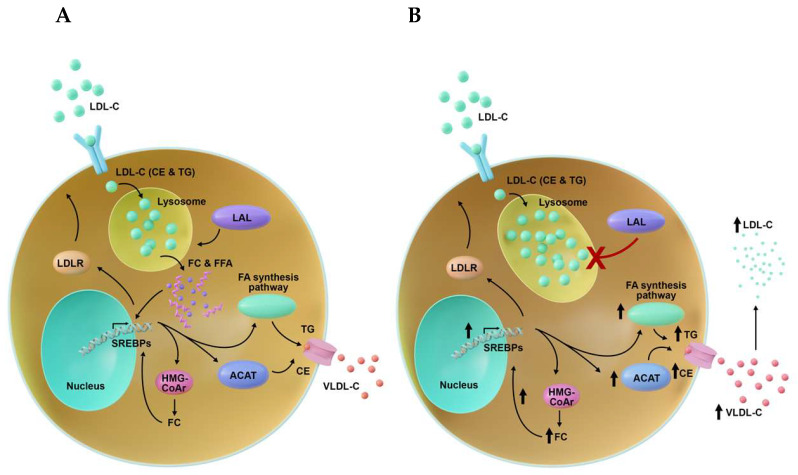
Consequence of LAL-D in lipid metabolism. (**A**). In a healthy cell, LDL-C is taken up by the LDL-C receptor and enters the lysosome. The LAL enzyme metabolizes the LDL-C to FC and FFA, which synthesizes triglycerides, CEs, and VLDL-C. (**B**). In a LAL-D cell, LDL-C, cholesterol esters, and triglycerides build up in the lysosome, causing the cells to balloon. The absence of physiologic lipid metabolism leads to lowered levels of FC and FAs, stimulating cholesterol biosynthesis and increasing endogenous production of LDL-C. ACAT, acyl-coenzyme A:cholesterol acyltransferase; CE, cholesteryl esters; FFA, free fatty acid; HMG-CoA, 3-hydroxy-3-methylglutaryl-coenzyme A; LAL-D, lysosomal acid lipase deficiency; LDL-C, low-density lipoprotein cholesterol; LDLR, low-density lipoprotein receptor; FC, free cholesterol; SREBP, sterol regulatory element-binding protein; TG, triglycerides; VLDL-C, very-low-density lipoprotein cholesterol.

LAL is necessary for the hydrolysis and release of cholesteryl esters and triglycerides from the lysosomes in the form of free cholesterol and free fatty acids (Figure 1) [7,8]. LAL-D is characterized by an accumulation of cholesteryl esters and triglycerides in the lysosomes of cells in the gastrointestinal tract and liver, causing these cells to balloon [4]. The absence of physiologic lipid metabolism leads to lowered levels of intracellular free cholesterol (FC) and fatty acids (FAs). Low levels of FC and FAs stimulate cholesterol biosynthesis and increase the production of low-density lipoprotein cholesterol (LDL-C) [4,7,9]. Lipid deposition and lipid abnormalities, such as increased release of triglycerides and very-low-density lipoprotein cholesterol (VLDL-C), as well as decreased formation of high-density lipoprotein cholesterol (HDL-C) result in the release of proinflammatory oxysterols and manifestations of disease [4,8,10]. Affected macrophages respond with activation and inflammation, promoting a systemic inflammatory response to atypical stimuli (e.g., excessive cholesterol) [6]. Systemic inflammation may predispose a patient with infantile-onset LAL-D to a secondary hemophagocytic lymphohistiocytosis, which can lead to an exceptionally rapid decline in the patient’s health [6,11,12,13].

Digestion and absorption of nutrients ingested in the diet, including lipids, take place in the small intestine [14]. These processes are impacted by lipid accumulation within the macrophages of the lamina propria in patients with LAL-D, derived both exogenously from diet and produced endogenously [14]. In the liver and spleen, lipid accumulation results in hepatomegaly, progressive liver dysfunction (indicated by increased transaminases), splenomegaly, and eventual liver failure. In the macrophages within the lymph nodes, lipid accumulation can lead to excessive mesenteric lymphadenopathy and inflammation [2,3,4], which can lead to blocked lymphatic outflow and intestinal and blood vessel blockage in the abdomen, and may be mistakenly interpreted as lymphatic malignancy [6,15].

Lipid accumulation in the lamina propria of the gastrointestinal tract results in local inflammation and distorted villi, resulting in reduced surface area and decreased availability of luminal digestive enzymes (e.g., proteases, lipase, disaccharidases), leading to decreased absorption of both macronutrients and micronutrients [1,14]. Consequently, infants with LAL-D present with growth failure and severe malabsorption due to frequent diarrhea, rapid dehydration, bacterial overgrowth, abdominal bloating with excessive gas, and dysmotility with persistent vomiting [16]. Abdominal distension due to hepatosplenomegaly, impaired intestinal passage, and lymphadenopathy can compromise venous blood flow and respiratory function and decrease tolerance of feed volumes [2,3,4]. Affected infants usually have intestinal failure, which greatly impacts the approach to successful management [6].

Mouse models of infantile-onset LAL-D provide evidence of the accumulation of lipids in the organs leading to inflammation [14,17,18]. LAL-knockout mice have reduced levels of HDL-C and elevated levels of LDL-C when exposed to typical rat chow. These mice then develop lipid-rich vacuoles in the intestinal lamina propria and impaired lipid absorption, as well as liver inflammation [14,17,18].

A multidisciplinary specialist team is necessary for managing this highly complex, multisystemic condition [19]. Historically, treatment options for infantile-onset LAL-D were limited. Hematopoietic stem cell transplantation (HSCT) demonstrated clinical efficacy, likely through two mechanisms: (1) healthy donor macrophages repopulate the recipient’s LAL-deficient, lipid-laden, vacuolated macrophages, and (2) the metabolic defects of other cells, such as hepatocytes, are corrected through LAL enzyme transfer from the donor hematopoietic stem cells [20]. However, before the advent of enzyme replacement therapy (ERT), most patients with infantile-onset LAL-D died during preparation for HSCT or within weeks or months after the procedure [5,19,21]. Today, treatment is multimodal, including both sebelipase alfa (KANUMA; Alexion, AstraZeneca Rare Disease), a human lysosomal acid lipase ERT that improves lipid metabolism [22], and nutritional management, comprising dietary substrate (lipid) reduction known as substrate reduction therapy, and management of gastrointestinal disturbances and poor growth. Together, ERT and nutritional management improve growth, gut, and liver function, reduce systemic inflammation, and improve quality of life and survival in patients with infantile-onset LAL-D and, owing to improved clinical conditions, may improve HSCT outcomes in those who undergo the procedure [6,19].

The published medical literature on the topic is limited and represents earlier guidance on nutritional management, which has changed over time as our knowledge and understanding of this ‘new’ phenotype of treated infantile-onset LAL-D has enhanced. Narrative, expert guidance on current nutritional management, including the choice of enteral feeds and supplementation, as well as clinically proven regimens for parenteral nutrition (PN), is lacking. This review aims to provide expert opinions based on the collective clinical experience of the authors, as physicians and specialist metabolic dietitians, to drive best-practice strategies and provide recommendations and guidance for the nutritional management of infantile-onset LAL-D. A plain language summary of this manuscript can be found in the Supplementary Materials.

## 2. Methods

Although this narrative review included a search of the literature via PubMed through September 2025 using keywords, including “lysosomal acid lipase deficiency”, “LAL-D”, “infantile-onset LAL-D”, “Wolman disease”, “nutritional management”, and “diet”, the recommendations herein are based on detailed long-term patient follow-up and the expert clinical experience and in-depth knowledge of the literature of the authors, who are physicians and specialist metabolic dietitians who support the care of patients with infantile-onset LAL-D.

## 3. Nutritional Management of LAL-D

Nutritional management of rapidly progressive LAL-D requires specialized feed formulas with an ultra-low-fat content (as close to zero as is achievable) with the objective of near-complete elimination of fat from the diet [6]. Breast milk and standard infant formula have a high fat content, with breast milk containing 3.5% to 4.5% fat, up to 98% of which is in the form of triglycerides; therefore, breast milk and normal infant formulas are contraindicated in this highly vulnerable population of infants [1,23,24]. Many therapeutic infant formulas, including high-energy infant formulas and those used to manage protein and/or disaccharide malabsorption or intolerance, are also not suitable because of their high lipid content [25,26]. Before diagnosis, infants are often switched from breast milk or standard infant formula to therapeutic infant formula to address symptoms such as weight loss, vomiting, and diarrhea without success, leading to continued symptoms and growth failure [27]. There is a direct link between lipid exposure and systemic inflammation; macrophages in the gastrointestinal tract of patients with rapidly progressive LAL-D are activated by exposure to lipids, significantly promoting inflammation [6,14]. Inflammation decreases nutrient absorption and causes damage to the intestinal tract over time (Table 1) [3,4,5,6,28].

Since the introduction of ERT, nutritional management for infantile-onset LAL-D has evolved. Initial empirical guidance on nutritional management was produced in conjunction with the first infant clinical trials of ERT [28,29] and was developed assuming that ERT would normalize gastrointestinal function in patients with rapidly progressive LAL-D. Historically, guidance included the use of lipid-restricted PN to promote growth and control gastrointestinal symptoms followed by a transition to a whole (intact) protein, low-fat formula with medium-chain triglyceride (MCT) supplementation (such as Monogen, Nutricia) [2], or an amino-acid-based, low long-chain triglyceride (LCT) feed with MCT when intact-protein-based feeds were not tolerated. MCTs, as medium-chain fatty acids (MCFAs), are absorbed from the small intestine and transported directly to the liver via the portal vein [30,31]. In the liver, MCFAs enter the mitochondria independent of the carnitine shuttle and then undergo rapid beta-oxidation to produce acetyl CoA, which is used in the tricarboxylic (Kreb’s) cycle, as well as in the synthesis of ketone bodies, for release into the bloodstream as a readily available source of energy for the body [30]. This contrasts with long-chain triglycerides, which require pancreatic lipase, mixed micelles, chylomicrons, and carnitine for absorption and metabolism [30].

### 3.1. Current Guidance on Parenteral and Enteral Nutritional Management

Current guidance now recognizes the requirement for and importance of long-term dietary substrate reduction therapy [6], minimizing total lipid intake. In infants, lipid intake should normally be restricted to <2 g/day until 6 months of age. After 6 months of age, lipid intake should continue to be as minimal as possible. Once solid foods are introduced, maintaining lipid intakes to <2 g/day may not be achievable. Therefore, a pragmatic goal is 0.5 g/kg per day up to a maximum of 5 g/day. At diagnosis, all patients require an assessment of nutritional intake, feed history, growth, and clinical symptoms, including gastrointestinal, to help guide appropriate nutritional management. Depending on the severity of gastrointestinal symptoms, this can range from modified minimal-lipid total parenteral nutrition (TPN) in those with the most severe symptoms, or an amino-acid-based, minimal-fat feed to an intact-protein, minimal-fat formula if there are no or minimal gastrointestinal symptoms (Table 2).

**Intact-protein**-**based feed.** If intact protein and lactose are tolerated, a specialized infant formula which has negligible lipid content may be used. For example, Low Fat Module (Nutricia) and basic-f (Milupa) both provide 2 g protein, 0.09 g fat (1.5% energy), and 11.5 or 11.6 g carbohydrate (of which 5.5 g is lactose) per 100 mL standard dilution [32,33]. Additional carbohydrate can be added as glucose polymer to increase the energy content [6]. However, formulas containing intact protein are rarely tolerated at presentation or diagnosis in clinically presenting cases unless the patient has few gastrointestinal symptoms. They will not be tolerated in patients with severe gastrointestinal involvement until there has been adequate gut recovery, which can take months to years [6].

**Amino acid-based feed.** Patients with moderate gastrointestinal symptoms may tolerate amino acid-based feeds (AABFs) with minimal lipid content. Availability of such commercial formulas is limited, as AABFs are generally high in lipids including long-chain triglycerides [6]. However, there are currently two commercially marketed AABFs available in some countries that have a minimal fat content: Vivonex T.E.N. (Nestle Health Science) provides 3.8 g amino acids per 100 mL standard dilution with 3% of energy from fat, and Tolerex (Nestle Health Science) provides 2.1 g amino acids per 100 mL standard dilution with 2% energy from fat (Table 3) [34,35]. Both are marketed for patients 3 years of age and older but have been used successfully in several cases of infant LAL-D. If these formulas are not available, a modular AABF with minimal lipid content will be required, composed of individual macronutrients, micronutrients, electrolytes, essential fatty acids, and long-chain polyunsaturated fatty acids that can be tailored to a patient as necessary. Additional sodium compared with normal requirements is usually required. An example of a modular AABF with minimal lipid content is provided in Table 4.

**Modified parenteral nutrition.** The majority of clinically presenting cases of infantile-onset LAL-D will require modified TPN or a combination of enteral nutrition (EN) and PN due to severe gastrointestinal symptoms, diarrhea in particular, as dehydration may occur even on minimal-fat AABF [6]. PN will allow for symptom relief, minimize inflammation, and provide adequate nutrition for catch-up growth and development. PN should include increased nitrogen and glucose to support growth but should not contain lipids, with the exception of fat-soluble vitamins. Most patients will also require a higher sodium intake (Table 2). Lipids should be avoided completely to start with, particularly until the patient is established on ERT, but may need to be considered in very small amounts as an addition to PN if adequate growth cannot be achieved on maximal nitrogen and glucose intakes (Table 2). If lipids are given temporarily, up to a maximum of 1 g/kg per day to help establish growth, a lipid emulsion with a higher percentage fat provided in the form of MCTs should be used, as these are metabolized in the liver; fatty acids should be balanced owing to the risk of inflammation [6,36]. Currently, in clinical practice, mixed lipid emulsions (such as soybean oil + MCT + olive oil + fish oil) are preferred to reduce pro-inflammatory effects and improve the immunologic profile. One such option is SMOFlipid (Fresenius Kabi), which is a mixture of soybean oil (omega-6 fatty acid), MCTs, olive oil (omega-9 fatty acid), and fish oil (omega-3 fatty acid), with balanced proportions of omega-3 and omega-6 fatty acids [6,37,38]. In contrast, purely soy-based lipid emulsions, such as Intralipid, are very high in omega-6 fatty acids only, which exacerbates inflammation [36,37].

Combining bespoke PN with small volumes of enteral feeding, with negligible lipid content is important to stimulate the gut mucosa, reduce the risk of atrophy, and improve immune function [6,39]. Oral intake should be encouraged when possible to preserve oral feeding skills, as many infants become averse. In infants with feeding tubes, it is important to stimulate the sucking reflex using a pacifier or finger stimulation. This practice helps maintain and strengthen the sucking reflex, promotes oral neuromuscular development, facilitates the coordination of breathing, sucking, and swallowing, and supports a smoother transition to oral feeding.

Once diarrhea and other gastrointestinal symptoms have eased, which can occur within a few days or weeks of starting modified PN, trophic enteral intake may be reintroduced via oral rehydration solution at 1 mL/kg per hour or as an equivalent daily volume divided into 2 to 3 hourly feeds given orally, alternatively as continuous tube and/or bolus feeds. Transition from modified PN to enteral feeds should be introduced slowly once growth has been reestablished and the gut has shown recovery (Table 3). The best choice of feed in this situation would be an AABF with minimal lipid content [6,19]. AABFs may be obtained as a commercially available product (e.g., Vivonex T.E.N. [Nestle Health Science], Tolerex [Nestle Health Science]) or as a modular elemental feed (Table 4) with the target of achieving a minimum of 4 g/kg per day of protein equivalent from free amino acids, when on full enteral feeds. Carbohydrate, in the form of glucose polymer, should be gradually increased (e.g., 1% increments) from approximately 10% to 20% concentration in feed (increasing from 10 up to 20 g carbohydrate per 100 mL of feed), with increases made as tolerated [6]. Higher concentrations of glucose are needed to compensate for the minimal levels of fat to meet energy requirements. Long-chain triglycerides should not be included, except in essential fatty acid supplements, and MCT should be used only if growth cannot be achieved by further maximizing carbohydrate and protein intake. Most patients will continue to require a significant sodium intake (potentially as high as >6 to 8 mmol/kg per day). Enteral feeds are usually given as continuous tube feeds initially to maximize tolerance. As enteral volumes are increased, PN should be decreased. In some cases, it may take from 1 to 6 months to establish total enteral feeding after ERT and dietary substrate reduction initiation [6]. Vomiting and diarrhea may persist, or even increase, but enteral feeding should continue unless there is consistent weight loss, decrease in mid-upper arm circumference (MUAC), or dehydration develops [6]. If gastroesophageal reflux develops, management may need to be considered.

Vitamin, mineral, and essential fatty acid status should also be taken into consideration. Profound deficiencies in the fat-soluble vitamins A, D, E, and K are common at the time of diagnosis, as is anemia [2,3,6,8,12]. Supplementation and monitoring will be required, including intravenous infusion of iron, since enteral iron is poorly absorbed in these cases [6]. Because of the minimal dietary lipid intake, essential fatty acids/long-chain polyunsaturated fatty acids will need supplementation (e.g., walnut oil, safflower oil, and KeyOmega [Vitaflo, Nestle Health Science]).

Once gastrointestinal function is restored and growth continues to be satisfactory in patients who required an AABF, the introduction of whole protein, minimal-fat feeds can be considered. This should be a gradual process (over 4 to 6 weeks); this time period could be reassessed if there are no problems with introduction. Suitable formulas for infants are Low Fat Module (Nutricia) and basic-f (Milupa); in older children, suitable feeds are based on fat-free milk or minimal-fat “juice”-type nutritional supplements. As knowledge of the nutritional needs of patients with LAL-D increases, verified web sites, such as “LAL-D Your Way” (https://laldyourway.com/) may become useful repositories of information for patients and caregivers.

### 3.2. Monitoring

Patient monitoring is essential across the lifespan. Growth should be carefully monitored during the initial phase of ERT and nutrition rehabilitation, with weight measured twice a week and length every 2 weeks. However, MUAC is a much better indicator of growth, as weight can be affected by other factors associated with LAL-D, such as increased liver and/or spleen size [6]. MUAC should be measured weekly while in patients while admitted to the hospital and at all follow-up reviews. Fat-soluble vitamins, essential fatty acids, and iron levels should also be monitored regularly. Blood glucose levels should be monitored when high-glucose TPN is being used [6]. Urinary and/or serum electrolytes should be monitored to assess for sodium depletion and should be repeated for as long as high sodium feeds are used.

For long-term treatment success, continued monitoring and adjustment of dietary management is necessary, as is ongoing dietetic education and support for the family and child. The introduction of weaning solids should not be delayed, but this requires detailed advice and education to ensure lipid intake remains restricted while the diet is made as palatable and varied as possible. Speech and/or occupational therapy may be needed to assess for and support oral aversion following PN and enteral feeding [40]. Once teeth have come in, regular dental visits should be prioritized, as a high carbohydrate diet increases the risk of cavities. Potential sources for lipid ingestion need to be considered, such as medications and essential fatty acid supplements. For example, propofol lipid emulsion is a potentially hidden lipid source, as can be fat-soluble vitamin and long-chain polyunsaturated fatty acid supplements: KeyOmega provides 0.8 g per 4 g sachet [41]. Noncompliance with dietary restriction will limit growth, worsen clinical symptoms and response to ERT; to ensure treatment success, continued monitoring of MUAC, weight, length/height, head circumference (using growth charts to interpret), and milestone achievement are essential [6].

## 4. Case Studies

### 4.1. Case Study 1: Diet Uncontrolled―2014

**Figure 2:**
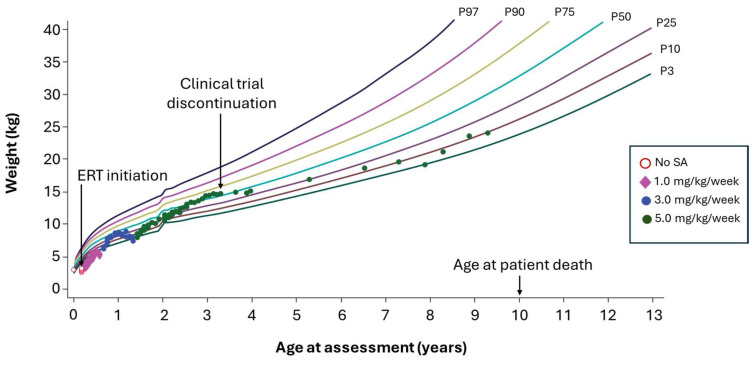
Case example 1. Moderate fat restriction and poor dietary compliance in a patient with 8 years of available follow-up. ERT, enzyme replacement therapy; SA, sebelipase alfa.

The patient was diagnosed at 20 days and presented at enrollment in a clinical trial at 2 months of age with systemic inflammation and liver failure and weight below the 3rd percentile. At 2.3 months, the patient began ERT with sebelipase alfa at 1 mg/kg per week. Diet from age 1 to 3 months included PN consisting of 1.5 g lipid/kg and 55 mL/kg Monogen. From age 3 to 6 months, the patient discontinued PN and received Monogen via NG tube and small amounts of low-fat solids. The patient’s weight percentile improved but began to decrease after approximately 6 months of treatment (Figure 2); the patient had delayed acceptance of oral foods. The sebelipase alfa dosage was raised to 3 mg/kg per week when the patient was 8 months of age, and weight again improved. At 1.5 years of age, the patient was hospitalized with abdominal pain and bowel impaction. Hydrolyzed whey MCT modular feed was introduced via NG tube, providing 1 g fat/kg per day; the oral diet was restricted to only very low-fat foods, but concerns were noted over parental compliance. The sebelipase alfa dosage was raised to 5 mg/kg per week, and the patient reached the 50th weight percentile by approximately 2.5 years.

From age 2 to 4 years, the patient received a fat-restricted modular AABF, promoting control of symptoms and improved growth. The NG tube was removed at age 4 years, with the patient taking the same very low-fat food as before but with no compliance concerns. At 5 years of age onward, patient weight decreased to the 25th percentile and then decreased steadily to the 3rd percentile by age 8 years with reduced MUAC, largely owing to inadequate macronutrient and micronutrient intake compounded by occasional suboptimal compliance with oral dietary fat restriction. The patient no longer accepted amino acid feed by mouth and refused NG tube placement; diet consisted of oral very low-fat foods. Eventually, the patient accepted fat-free nutritional drinks when growth failure affected her wellbeing. This patient died at age 10 years from complications of HSCT.

### 4.2. Case Study 2: Diet Controlled with Difficulty—2017

The patient was admitted to the hospital at 2 weeks of age, presenting with vomiting, diarrhea, and poor weight gain. The patient was started on elemental formula (Neocate). At 2 months and 10 days of age, the patient was transferred to a referral hospital, presenting with malnutrition, persistent abdominal distension, and hepatosplenomegaly and weight for age below the 3rd percentile. After LAL-D diagnosis, the patient began ERT with sebelipase alfa and continued with the elemental formula. Transaminase levels improved after ERT initiation and the patient showed rapid improvement in hepatosplenomegaly and hypertransaminasemia, but severe malabsorption and poor weight gain persisted. A low-fat, whole protein formula (Monogen) was not tolerated by the patient due to protein intolerance secondary to intestinal damage; therefore, the patient continued on the elemental formula despite its higher lipid content.

**Figure 3:**
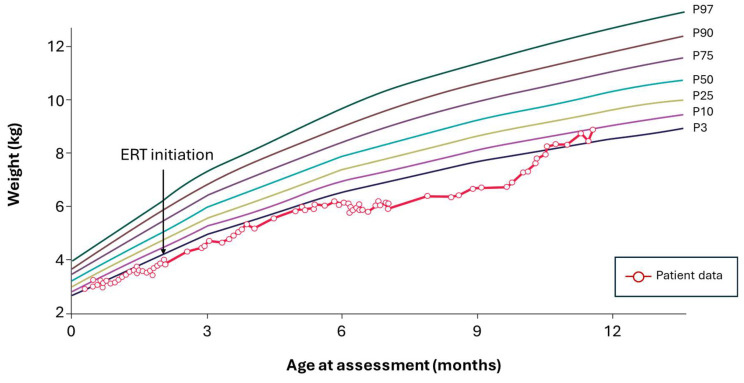
Case example 2. Early diagnosis and difficult nutritional stabilization. ERT, enzyme replacement therapy.

At 10 months of age, the patient was well below the 3rd percentile in weight for age and continued to be hospitalized. The patient’s diet was switched to a PN and a minimal-fat elemental modular feed based on a formula developed by the metabolic dietitians at The Willink Biochemical Genetics Unit (Manchester, UK). This recipe contained essential amino acids, glucose, MCT emulsion, essential fatty acids, oral rehydration solution, and micronutrients, providing 4 g protein, approximately 20 g carbohydrate, and 1 g fat per kg per day (as MCT, with minimal LCTs from essential fatty acids). After the switch to this modular feed, the patient began to improve, and ferritin levels stabilized. Gastrointestinal symptoms improved, and rapid weight gain was observed. The patient was discharged from hospital at 12 months of age, with weight for age over the 3rd percentile (Figure 3). At 33 months, whole protein feeds were introduced using a low-fat formula (Monogen), which was later replaced with regular skimmed milk. At the time of writing, the patient is 8 years old, he follows a low-lipid diet by mouth, and his weight and height are in the 10th percentile of normal for his age.

### 4.3. Case Study 3: Diet Controlled—2025

**Figure 4:**
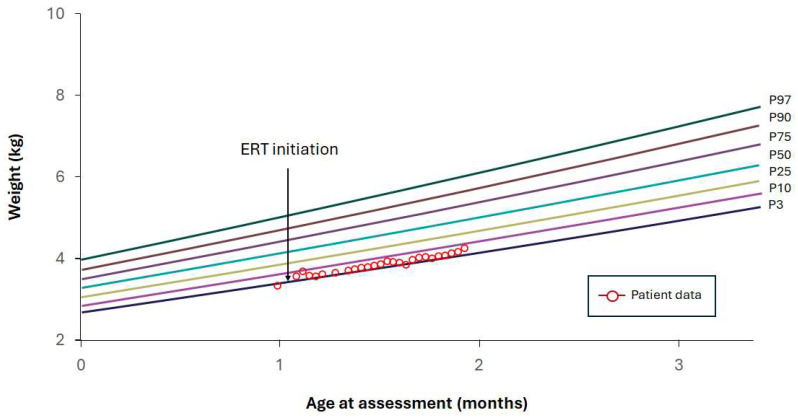
Case example 3. Early diagnosis and nutritional stabilization. ERT, enzyme replacement therapy.

The patient, a full-term male newborn with normal birth weight, presented with vomiting and abdominal distension from the first days of life. He was admitted to the hospital due to failure to thrive and persistent abdominal distension. Abdominal x-ray showed bilateral adrenal calcifications. The patient was diagnosed with rapidly progressive LAL-D at 1 month of age and was immediately started on a low-fat elemental modular formula. The following day, at 1 month and 1 day of age, ERT with sebelipase alfa was initiated at a dose of 3 mg/kg twice weekly for the first 3 weeks, followed by 3 mg/kg once weekly thereafter. Enteral nutrition was initially administered via continuous feeding through a transpyloric tube for the first 14 days, followed by nasogastric tube feeding during the subsequent week. Oral feeding was introduced at 1 month and 22 days of age. At the time of diagnosis, the patient’s weight-for-age was below the 3rd percentile, and MUAC was 9 cm. By the time of hospital discharge, weight-for-age had reached the 3rd percentile, and MUAC was 11 cm (Figure 4). The patient was discharged at 1 month and 29 days of age, fully tolerating oral feeds with a low-fat elemental modular formula prepared at home by the parents (Table 5). At the time of writing, the patient was 8 months old and continued to be closely followed.

## 5. Discussion

This review provides guidance on some of the many factors associated with nutritional care of the patient with infantile-onset LAL-D. There have been, as of now, no established, published guidelines on dietary management in this patient population, although recommended dietary interventions and management have evolved over time, particularly from those centers managing significant numbers of cases. Previous recommendations included a low-fat diet based on MCTs, and the assumption that most patients would need a period of TPN. This review is intended to illuminate the ongoing evolution of the management approach to LAL-D, which as of yet remains largely unpublished. The care of patients with infantile-onset LAL-D must include combined management with ERT and significant dietary substrate reduction with minimal lipid intake through diet modification whether being fed parenterally or enterally [6]. Nutritional interventions must be individualized according to the patient’s specific needs, which may change with time and clinical state.

Early diagnosis, treatment, and sustained adherence to a minimal lipid diet are the key to the management of LAL-D, as continued inflammation from lipid exposure and accumulation leads to gut and other organ damage, as well as poor growth [6]. LAL-D can be diagnosed using dried blood spot samples, raising the potential benefit for newborn screening [6,42]. LAL-D fulfills the Wilson–Jungner criteria for newborn screening [43]. Screening could mitigate the delays in diagnosis that result from the symptoms of LAL-D mimicking those of other, more common disorders, including cow’s milk protein intolerance, hemophagocytic lymphohistiocytosis, and metabolic-associated fatty liver disease [6,42,44]. Newborn screening pilot studies are ongoing in multiple countries [45,46,47]. In patients diagnosed early based on family history who begin ERT and follow a minimal-fat diet very early, normal weight and growth can be established and maintained throughout childhood. Lifestyle changes initiated early in life are better accepted, and lead to higher adherence to dietary treatment [42,48]. Therefore, patients with a family history of LAL-D (e.g., an affected sibling) should be tested as soon as possible and started on a minimal lipid formula from birth until it is known whether they are affected [42,48].

Frequent monitoring is needed for infants with LAL-D as treatment and dietary changes are initiated, ideally performed daily during the initial inpatient stabilization and then every 1 to 2 weeks over the first 6 months. As the patient ages, time between visits can lengthen to every 6 months [6]. General guidance calls for long-term adherence to a minimal-fat diet through the lifespan of patients with LAL-D [19]. While children on therapeutic diets from a young age do not necessarily find them unpalatable or unsatiating, adherence can become more difficult with increasing age and independence. Adolescents, in particular, may find it difficult to follow a more restrictive diet than their peers. For this reason, continued patient and caregiver education and care [6] via a multidisciplinary team that includes metabolic pediatricians, registered metabolic dietitians, and nurse specialists, as well as psychological support, are keys to continued treatment success [44].

## Figures and Tables

**Table 1:** Impact of LAL-D on the GI system [3,4,5,6,28].

Altered GI Function	Associated GI Signs and Symptoms
Fat, disaccharide, and protein malabsorption [6,28] Dysmotility [6] Reduced absorptive area [6] Reduced gut enzymes and nutrient digestion [6] Deficiency of fat-soluble vitamins and essential fatty acids [6] Inability to tolerate enteral feeds [6] Poor feed volume tolerance [6]	Rapid dehydration [6] Gallbladder dysfunction [3,4] Abdominal bloating and gas [6] Hypoalbuminemia [5,28] Abdominal and epigastric pain [3,4] Failure to thrive [3,4,5,28] Emesis [28] Growth failure [28] Frequent diarrhea [4,28] Steatorrhea [3,4,5] Cachexia [3,4] Hepatomegaly [3,4]

GI, gastrointestinal; LAL-D, lysosomal acid lipase deficiency.

**Table 2:** Suggested nutritional management for infants with LAL-D.

Nutritional Concern	Nutritional Intervention	Choice of Product/Feed Supplement
Fat malabsorption/ utilization	Low total fat formula	Low Fat Module (Nutricia), basic-f (Milupa)
Protein malabsorption	High protein: ≥4 g/kg per day Elemental low-fat feed	Low fat, amino acid-based formula (e.g., Vivonex T.E.N. or Tolerex from Nestle Health Science) Modular feed of - Amino acids - Glucose polymer - Micronutrients - Electrolytes - Essential fatty acids
Poor weight gain	Increase calories Concentrate formula with additional carbohydrate and consider additional MCT	Glucose polymer (e.g., Vitajoule from Vitaflo)
Poor volume tolerance	Decrease feed volume, increase frequency Continuous pump feed	
Failure to tolerate enteral feed	Modified PN Minimal fat Increased protein High glucose	TPN should be lipid free SMOFlipid if calories cannot be increased in other ways, to no more than 1 g/kg per day until adequate growth is established
Fat soluble vitamin deficiency	Supplement	Supplementation of vitamins A, D, E, and K (if there is prolonged blood clotting time)
Essential fatty acids and long-chain polyunsaturated fatty acid deficiency	Supplement, normal-to-increased requirements	KeyOmega (Vitaflo, Nestle Health Science), walnut oil, safflower oil
Carbohydrates	Monosaccharide sources	Glucose polymer
Sodium	Increased, 6–8 mmol sodium/kg per day adjusted to maintain urinary sodium >20 mmol/L and sodium: potassium ratio ≥ 2:1	Table salt Oral rehydration solutions
Iron	Provide at least normal requirements in feeds; additional iron supplementation may be required	Enteral iron supplements; IV iron may be required if not tolerated/absorbed

IV, intravenous; MCT, medium-chain triglyceride; PN, parenteral nutrition; SMOF, soy, medium-chain triglycerides, olive and fish oil; TPN, total parenteral nutrition.

**Table 3:** Commercially available amino acid, monosaccharide, and minimal LCT formulas (available in some countries).

Per 100 mL Standard Dilution	Energy, kcal (kJ)	Protein, g	CHO, g	LCT, g	MCT, g	Na, ^b^ mmol	K, mmol
Vivonex T.E.N. ^a^ (Nestle Health Science)	100 (420)	3.83	20.6	0.3	0	2.7	2.4
% Energy		15	82	3			
Tolerex ^a^ (Nestle Health Science)	100 (420)	2.05	22.6	0.2	0	2.2	3.0
% Energy		8	90	2			

^a^ Requires essential fatty acid supplementation. ^b^ May require additional sodium. LCT, long-chain triglycerides; MCT, medium-chain triglycerides; T.E.N., total enteral nutrition.

**Table 4:** Amino acid, monosaccharide (glucose polymer), and minimal LCT feed without MCT nutritional composition (per 100 mL, based on full feeds of 100 mL/kg per day).

	Amount, g	Energy, kcal (kJ)	Protein, g	CHO, g	LCT, g	Na, mmol	K, mmol
Complete amino acid mix (Nutricia [Danone])	5	16 (67)	4.1	0	0	0	0
Glucose polymer (e.g., Vitajoule [Vitaflo])	18	68 (284)	0	17.1	0	0	0
Oral rehydration solution (e.g., Dioralyte powder [Sanofi])	2.5	7 (29)	0	1.8	0	6	2
Walnut oil (for essential fatty acids) ^a^	0.15 mL	1 (4)	0	0	0.14		
Long-chain polyunsaturated fatty acids (e.g., KeyOmega [Vitaflo, Nestle Health Science] ^b^)	4 ^b^	2 (8)	0	0.3	0.8	0.06	0
Vitamin/mineral supplement (e.g., Pediatric Seravit [Nutricia] ^c^)	5	1 (4)	0	1.3	0	0	0
+Water to 100 mL	Per 100 mL	95 (398)	4.1	20.5	0.94	6.1	2
% Energy contribution			17.3	73.8	8.9		

^a^ Walnut oil: 0.1 mL per 56 kcals (235 kJ) to provide UK-suggested requirement for essential fatty acids. ^b^ Amount per 24 h; can be added to 1 feed per day or to the full 24 h volume. One sachet (4 g) of KeyOmega provides 200 mg arachidonic acid and 100 mg DHA. ^c^ Suggested 24 h daily dosing of Pediatric Seravit by age is 0–6 months, 10 g; 6–12 months, 15 g; 1–3 years, 15 g. DHA, docosahexanoic acid; LCT, long-chain triglycerides; MCT, medium-chain triglycerides.

**Table 5:** Modular formula recipe and preparation instructions.

Ingredient	Quantity
Suero Oral 60 (electrolyte solution)	19.5 g
Dextrinomaltose (glucose)	127 g
Amino acid mix	25 g
Pediatric Seravit (vitamin/mineral supplement)	13 g
MCT oil/water emulsion (lipid)	2.5 mL MCT oil + 2.5 mL water = 5 mL
KeyOmega (essential fatty acids) [Vitaflo, Nestle Health Science]	¾ sachet = 3 g
Water	To a final volume of 750 mL
**Preparation Steps**	
**Weigh** the Suero Oral 60, dextrinomaltose, Amino acid mix, and Pediatric Seravit. Transfer the weighed powders into a large container (e.g., a jug). Gradually add water up to approximately 400 mL while stirring continuously until a homogeneous mixture is achieved. **Separately**, prepare the fat emulsion in a 10–20 mL syringe by mixing 2.5 mL of MCT oil with 2.5 mL of water. Once this solution is emulsified, add 3 g of KeyOmega (¾ sachet) and mix thoroughly. **Combine** the aqueous mixture (Step 1) with the lipid emulsion (Step 2). Mix well and make up the final volume to 750 mL with water. **Transfer** the prepared formula into a plastic bottle. Protect from light. **Storage and Shelf-life:** Store refrigerated. Use within 24 hours.	

MCT, medium-chain triglycerides.

## Data Availability

Alexion, AstraZeneca Rare Disease will consider requests for disclosure of clinical study participant-level data provided that participant privacy is assured through methods like data de-identification, pseudonymization, or anonymization (as required by applicable law), and if such disclosure was included in the relevant study informed consent form or similar documentation. Qualified academic investigators may request participant-level clinical data and supporting documents (statistical analysis plan and protocol) pertaining to Alexion-sponsored studies. Further details regarding data availability and instructions for requesting information are available in the Alexion Clinical Trials Disclosure and Transparency Policy at https://www.alexionclinicaltrialtransparency.com/data-requests/.

## Supplementary Materials

The following supporting information can be downloaded at: https://www.mdpi.com/article/10.3390/nu18020233/s1, A plain language summary.
