# Supplementary material for: Best Practices for the Nutritional Management of Infantile-Onset Lysosomal Acid Lipase Deficiency: A Case-Based Discussion
Source: Nutrients. 2026 Jan 12;18(2):233. doi: 10.3390/nu18020233 (PMC12845284; doi:10.3390/nu18020233)
Supplement: Supplementary file 1 [file nutrients-18-00233-s001.zip › nutrients-3962230-supplementary.pdf]

Plain Language Summary

Infant-onset lysosomal acid lipase deficiency (LAL-D), also known as Wolman disease, is a very rare and life-threatening inherited condition. Patients with LAL-D are missing an enzyme called lysosomal acid lipase (LAL). Without this enzyme, fat builds up in the cells of the body. This causes serious health problems in infants, such as vomiting, diarrhea, large liver and spleen, and poor growth. It can become worse quickly, and most infants may die within the first year without treatment.

There are two parts to LAL-D treatment. First, the missing enzyme is replaced with a medication called sebelipase alfa, which helps reduce the fat build-up in the body. Second, infants must reduce overall fat intake by following a special diet that includes only specific types of fat that do not build up. This diet will also help manage inflammation, diarrhea, and vomiting. Infants must be monitored to make sure levels of vitamins and minerals are within recommended ranges. Together, these treatments can improve health, growth, and long-term quality of life.

The goal of this review is to provide updated best practice guidance on how to manage diet in infant-onset LAL-D. The guidance we provide herein is based on knowledge gained from years of treating these infants and developing the best diets and nutritional therapies to improve quality of life.

This review highlights the importance of combining both medical and dietary treatments. It also shows the need for a specialized care team. Future guidelines should reflect these best practices.
